# Current Perspectives on the Role of Matrix Metalloproteinases in the Pathogenesis of Basal Cell Carcinoma

**DOI:** 10.3390/biom11060903

**Published:** 2021-06-17

**Authors:** Mircea Tampa, Simona Roxana Georgescu, Madalina Irina Mitran, Cristina Iulia Mitran, Clara Matei, Ana Caruntu, Cristian Scheau, Ilinca Nicolae, Andreea Matei, Constantin Caruntu, Carolina Constantin, Monica Neagu

**Affiliations:** 1 Department of Dermatology, Carol Davila University of Medicine and Pharmacy, 020021 Bucharest, Romania; tampa_mircea@yahoo.com (M.T.); matei_clara@yahoo.com (C.M.); 2 Department of Dermatology, Victor Babes Clinical Hospital for Infectious Diseases, 030303 Bucharest, Romania; drnicolaei@yahoo.ro; 3 Department of Microbiology, Carol Davila University of Medicine and Pharmacy, 020021 Bucharest, Romania; madalina.irina.mitran@gmail.com (M.I.M.); cristina.iulia.mitran@gmail.com (C.I.M.); 4 Department of Oral and Maxillofacial Surgery, “Carol Davila” Central Military Emergency Hospital, 010825 Bucharest, Romania; 5 Faculty of Dental Medicine, Titu Maiorescu University, 031593 Bucharest, Romania; 6 Department of Physiology, Carol Davila University of Medicine and Pharmacy, 050474 Bucharest, Romania; cristian.scheau@umfcd.ro (C.S.); andreea.matei@drd.umfcd.ro (A.M.); costin.caruntu@gmail.com (C.C.); 7 Department of Dermatology, Prof. N.C. Paulescu National Institute of Diabetes, Nutrition and Metabolic Diseases, 011233 Bucharest, Romania; 8 Immunology Department, Victor Babes National Institute of Pathology, 050096 Bucharest, Romania; caroconstantin@gmail.com (C.C.); neagu.monica@gmail.com (M.N.); 9 Department of Pathology, Colentina University Hospital, Bucharest 020125, Romania; 10 Faculty of Biology, University of Bucharest, Bucharest 76201, Romania

**Keywords:** BCC, MMP, TIMP, invasion, tumor progression

## Abstract

Basal cell carcinoma (BCC) is the most common skin malignancy, which rarely metastasizes but has a great ability to infiltrate and invade the surrounding tissues. One of the molecular players involved in the metastatic process are matrix metalloproteinases (MMPs). MMPs are enzymes that can degrade various components of the extracellular matrix. In the skin, the expression of MMPs is increased in response to various stimuli, including ultraviolet (UV) radiation, one of the main factors involved in the development of BCC. By modulating various processes that are linked to tumor growth, such as invasion and angiogenesis, MMPs have been associated with UV-related carcinogenesis. The sources of MMPs are multiple, as they can be released by both neoplastic and tumor microenvironment cells. Inhibiting the action of MMPs could be a useful therapeutic option in BCC management. In this review that reunites the latest advances in this domain, we discuss the role of MMPs in the pathogenesis and evolution of BCC, as molecules involved in tumor aggressiveness and risk of recurrence, in order to offer a fresh and updated perspective on this field.

## 1. Introduction

Basal cell carcinoma (BCC) is the most common form of skin cancer in humans, displaying a worldwide increase in incidence. BCC may be considered the result of a complex interaction between genetic and environmental factors, with exposure to ultraviolet (UV) light being a key player in its pathogenesis. The incidence of BCC starts to increase within the fourth decade of life, while young people are rarely affected. An exception to this tendency is constituted by the patients with either genodermatoses (such as xeroderma pigmentosum, Gorlin-Goltz syndrome, Bazex or Rombo syndrome) or different degrees of immunosuppression [1,2]. BCC most commonly appears on the face in individuals with fair skin; other possible locations are the trunk and extremities. According to some recent theories, BCC originates in hair follicles; therefore, it is rarely, if ever, diagnosed on non-hair-bearing sites such as the mucous membranes (e.g. oral or genital mucosa) [3].

In spite of the fact that it is a slow-growing tumor rarely displaying local invasiveness and metastasis, BCC causes, due to its ability to invade and infiltrate the surrounding tissue, considerable morbidity; altogether, due to its high incidence, it represents an important public health issue [1,2]. Hence, numerous efforts are made to discover new noninvasive diagnostic techniques [4,5] and new candidate molecules that can be used as both biomarkers of progression and future therapy targets in BCC.

The pathogenesis of BCC is complex and incompletely deciphered. Matrix metalloproteinases (MMPs) create a suitable microenvironment for tumor development, representing key molecules in tumor progression [6]. In this review, we aim to assemble novel data on the involvement of MMPs in the pathogenesis and progression of BCC and underline the role of these proteolytic enzymes in tumor aggressiveness, the risk of recurrence and as a valuable source for scouting new BCC therapeutic approaches.

## 2. MMPs as Molecular Promoters in Carcinogenesis

MMPs are members of the metzincin protease superfamily of zinc-endopeptidases and have traditionally been described as molecules that primarily degrade extracellular matrix (ECM) proteins [7]. Other members of the superfamily are A disintegrin and metalloproteinases (ADAMs) and ADAMs with thrombospondin motifs (ADAMTSs), which present, in their structure, a conserved methionine residue adjacent to the active site [7]. Nowadays, it is well-known that MMPs act on a wide range of substrates, including membrane receptors, cytokines, growth factors, signaling molecules and ligands [8]. Recent experimental in vivo studies have suggested that the main substrates of MMPs are nonmatrix molecules [8,9,10,11]. Therefore, MMPs can be considered as cell signal regulators rather than as destructive enzymes [8,9]. In the human body, MMPs are synthesized by numerous cellular types, such as fibroblasts, macrophages, endothelial cells, vascular smooth muscle, osteoblasts, etc. [12,13]. MMPs are grouped into several classes depending on the organization mode of their structural domains (Table 1). The structure of MMPs includes a propeptide, a metalloproteinase domain with catalytic action, a linker peptide of variable length and a hemopexin domain [12].

MMPs can be grouped into soluble MMPs and membrane-bound MT-MMPs. Soluble MMPs are released as inactive zymogens and, subsequently, are activated in the extracellular space by other MMPs and/or other proteases. MT-MMPs display a basic amino acid motif of RX(K/R)R at the C-terminal site of their prodomain, which is cleaved by proprotein convertases (PCs) (e.g., furin), resulting in the activation of the enzyme [14,15,16]. Their activity is modulated by general protease inhibitors, including α2-macroglobulin, and by specific inhibitors, such as tissue inhibitors of metalloproteinases (TIMPs) [17].

Recent research has revealed new in vivo substrates for these proteases. MMPs are involved in angiogenesis by modulating the bioavailability of angiogenic factors that are sequestered by the ECM or the basement membrane. For example, VEGF is mobilized by MMP-1, MMP-3, MMP-7, MMP-9, MMP-16 and MMP-19 through the cleavage of VEGF-binding ECM proteins [8]. Another interesting observation is that MMP-14 overexpression is associated with increased VEGF-A transcriptional activation [18]. MMPs increase cell migration both by degrading the ECM that impedes cell motility and by breaking down the proteins of adherens junctions, maintaining cellular cohesion [19]. In this case, E-cadherin is an important target. Another MMP substrate related to cell migration is the γ2 chain of laminin-5 [20]. MMPs also participate in the inflammatory process. The action of MMPs on the ECM components leads to the release of chemotactic molecules. For example, the fragments of elastin that results through MMP-12 cleavage may represent a chemoattractant factor for the influx of macrophages. In addition, MMP-12 can act on the serine protease inhibitor α1-PI, a non-ECM protein, resulting in components that act as chemoattractants for neutrophils [8]. The discovery of new substrates for MMPs offers novel perspectives on the role of MMPs in disease pathogenesis. MMPs are deeply involved in cell differentiation and proliferation, as well as in apoptosis, angiogenesis and the immune response [12,13].

The increased expression of MMPs in tumor cells and adjacent tumor tissue has been associated with a more aggressive tumor behavior [21,22,23]. The sources of MMPs are multiple; therefore, MMPs can be released by stromal cells, tumor cells or circulating cells [24]. The degradation of the ECM and basement membrane are important events in tumor invasion and metastasis [25]. Certain proteins in the ECM structure, such as fibronectin and laminin, promote cell migration and angiogenesis [26]. Basement membrane (BM) disruption is a key event in tumor invasion. Cells employ various mechanisms to cross the basement membrane; some of these events still remain incompletely elucidated. Cells may use protease-dependent or -independent invasion programs. Protease-dependent transmigration relies on the activity of membrane-type matrix metalloproteinases [27]. Using COS cells (i.e., an epithelial cell type that displays no BM degradative or invasive activity), it has been shown that MMP-2, MMP-3, MMP-7, MMP-9, MMP-11 and MMP-13 do not enable COS cells to degrade the BM. Conversely MMP-14, MMP-15 and MMP-16 endow COS cells with the ability to remodel the BM [27]. Collagens are most abundant in the ECM. The degradation of type I and III collagens is catalyzed by MMP-1, MMP-8, MMP-13, MMP-14 and MMP-16. MMP-2 degrades the solubilized monomers of collagens I, II and III [28]. Regarding MMP-2 and MMP-9, Rowe and Weiss point out that MMP-2 or MMP-9 can degrade in vitro type IV collagen; however, this ability is limited in vivo [27]. It should be considered that some substrates identified in vitro do not necessarily predict the activity of these enzymes in vivo; therefore, the studies performed in vivo should represent the basis for understanding the physio/pathological roles of MMPs.

Moreover, MMPs modulate several signaling pathways that contribute to tumor progression, activating cell signaling molecules, such as focal adhesion kinase (FAK), mitogen-activated protein kinase (MAPK), Rous sarcoma oncogene (SRC), rat sarcoma viral oncogene homologous (RAS) and phosphatidylinositol 3-kinase (PI3K) [24]. It is known that MMPs are involved in all stages of tumor progression and that they modulate crucial signaling pathways [29,30]. For example TIMP-2 binds to MMP-14 on the tumor cell surface, and this binding induces cell proliferation mediated through the subsequent activation of extracellular-regulated kinases (ERK) 1/2 [31]. Similarly, MMP-7 and ADAM10 induce antiapoptotic signaling events in cancer cells through the cleavage of the Fas ligand from the cell’s surface [32]. In other types of cancers, MMPs in the absence of their inhibitors activate canonical Wnt-signaling pathways, favoring the invasive capacity of cells [33].

In addition, MMPs can induce the shedding of the transmembrane precursors of the growth factors and growth factor-binding proteins (GF-BP). When MMPs degrade the insulin-like growth factor-binding protein (IGF-BP), IGFs are released [34]. Fibroblast growth factors are released when the basement membrane-specific heparan sulfate proteoglycan core protein is cleaved by MMPs. Through these processes, MMPs contribute to a pro-tumoral milieu [35].

MMPs also indirectly participate in the regulation of proliferative signals by integrins [26]. MMPs can be considered angiomodulators, as they are involved in the activation of proangiogenic factors and participate in the formation of new vessels, important structures sustaining tumor growth and progression [7]. Thus, many recent studies have highlighted that MMPs contribute to the main carcinogenesis steps, such as tumor progression, angiogenesis, invasion and metastasis; therefore, these biomolecules may also be regarded as potential target molecules in the prevention and treatment of neoplasms [36,37].

**Table 1:** MMP classifications and their substrates.

Groups	Substrates and Targets	MMPs as Activators for Other MMPs
**Collagenases** [8,9,12,38,39]		
MMP-1 (collagenase-1)	type I, II, III, VII, VIII, X and XI collagens [40,41], gelatin, nidogen [42], casein, aggrecan [43], perlecan, serpins, tenascin-C [44], versican, vitronectin, fibronectin, L-selectin, ovostatin, myelin basic protein, SDF-1 [45], pentraxin-3 [46], IGFBP [47], TNF precursor [48], VEGF-binding ECM proteins [49]	MMP-1 activates pro-MMP-2 [50] and pro- MMP-9 [51]
MMP-8 (collagenase-2)	type I, II, III, V, VII, VIII, and X collagens [52], gelatin, aggrecan [53], elastin, laminin [54], nidogen, fibronectin [55], ovostatin	MMP-8 activates pro-MMP-8
MMP-13 (collagenase-3)	type I-IV, IX, X, and XIV collagens [56,57,58], gelatin, plasminogen, fibronectin [58], osteonectin, aggrecan [59], perlecan [35], laminin, tenascin [58], casein	MMP-13 activates pro-MMP-2 and pro-MMP-9 [60]
MMP-18 (collagenase-4)	type I-III collagens, gelatin	
**Gelatinases** [8,9,11,12,46,61]		
MMP-2 (gelatinase A)	gelatin, type I-V, VII, X and XI collagens [40,62,63], elastin [63], aggrecan, laminin [64], fibronectin, nidogen, versican [51], tenascin, vitronectin, myelin basic protein, IGFBP-5 [65], follistatin-like 1 protein [66], follistatin-like 3 protein [46], mHB-EGF, CCL7/MCP-3 [67], CX3CL1/fractalkine, galectin-1, galectin-3 [68], transglutaminase [69], osteopontin, big endothelin-1 [70], TNF precursor, TGF beta, thrombospondin-2 and pyruvate kinase M1/M2 [71]	
MMP-9 (gelatinase B)	gelatin, type IV, V, VII, X and XIV collagens [40,63,72,73], aggrecan [43], elastin [63], fibronectin, laminin, nidogen, versican [51], decorin, myelin basic protein, casein, vitronectin, cytokines, chemokines, mHB-EGF [74], interleukin-8 [75], galectin-3 [68], interleukin-2 receptor-α [76], GCP-2/LIX [77], IGFBP [71], TNF precursor, TGF beta, VEGF-binding ECM proteins [49], thrombospondin-2 and pyruvate kinase M1/M2 [71]	
**Stromelysins** [8,9,12,26,78,79]		
MMP-3 (stromelysin-1)	type I-V [63] and IX-XI collagens, gelatin, aggrecan [80], ovostatin, nidogen [81], laminin, elastin, casein [82], osteonectin [60], decorin [44], fibronectin, perlecan [35], proteoglycans, versican [51], tenascin, myelin basic protein, osteopontin, plasminogen [83], IGFBP-3 [84], TNF precursor, VEGF-binding ECM proteins [49]	MMP-3 activates pro-MMP-1 [85], pro-MMP-8, pro-MMP-13 [86] and gelatinases [87,88]
MMP-10 (stromelysin-2)	type III-V [63,89], IX and X collagens, gelatin [86], aggrecan [80], fibronectin, casein [82], elastin [63], laminin, nidogen, proteoglycans, fibrilin-10	MMP-10 activates pro-MMP-1 [82], pro-MMP-8 [90] and pro-MMP-10
MMP-11 (stromelysin-3)	gelatin, fibronectin [91,92], aggrecan, laminin receptor [78]	
**Matrilysins** [8,9,12,61]		
MMP-7 (matrilysin-1)	type IV and X collagens [41,93], gelatin [93], aggrecan [43], decorin [94], elastin [63], entactin [73], casein [93], transferrin [95], fibronectin [93], laminin [93], plasminogen [96], vitronectin, tenascin [97], myelin, proteoglycans, β4-integrin, mHB-EGF [98], E-cadherin [19], osteopontin, syndecan [99], FasL [100]	MMP-7 activates pro-MMP-2 [101], pro-MMP-7 and pro-MMP-9 [102]
MMP-26 (matrilysin-2)	type IV collagen [103], gelatin [103], fibronectin [103], fibrinogen, vitronectin [104], casein, α2-macroglobulin	MMP-26 activates pro-MMP-2 and pro-MMP-9
**Trans-membrane** [8,9,12,46,105]		
MMP-14 (MT1-MMP)	type I-III collagens [106,107], gelatin, casein, fibronectin, laminin, nidogen [107], aggrecan, elastin, fibrin, perlecan, tenascin, vitronectin and proteoglycans [106,107,108], fibrilin-1, α2-macroglobulin [106], dickkopf-1 and cysteine-rich motor neuron-1 [109], galectin-1 [110], galectin-3 [111], syndecan-1, follistatin-like 3 protein [46], cyclophilin A [46], transglutaminase [112], pentraxin-3 [46]	MMP-14 activates pro-MMP-2 [113], pro-MMP-8 and pro-MMP-13 [114]
MMP-15 (MT2-MMP)	type I collagen, gelatin, fibronectin [107], laminin [107], aggrecan, perlecan [107], nidogen [107], tenascin, vitronectin, transglutaminase [112]	MMP-15 activates pro-MMP-2 [115] and pro-MMP-13
MMP-16 (MT3-MMP)	type I and III collagens [116], gelatin, casein [117], fibronectin, aggrecan, laminin, perlecan, vitronectin, syndecan [118], transglutaminase [112], VEGF-binding ECM proteins [49]	MMP-16 activates pro-MMP-2 [115], pro-MMP-9 and pro-MMP-13
MMP-24 (MT5-MMP)	gelatin, fibronectin [119], proteoglycans [119], N-cadherin [120]	MMP-24 activates pro-MMP-2 [121] and pro-MMP-13
**GP1-anchored** [8,9,11,12,26,122,123]		
MMP-17 (MT4-MMP)	fibrinogen [124], fibrin [124], gelatin [125], TNF precursor	
MMP-25 (MT6-MMP)	type IV collagen, proteoglycans, gelatin [126], fibronectin [127], vimentin [122], cystatin C [122], galectin-1 [122]	MMP-25 activates pro-MMP-2 [128]
**Other enzymes** [8,9,12,38]		
MMP-12 (macrophage metalloelastase)	type I, IV [129] and V collagens, gelatin [129], elastin [130], fibronectin [129], laminin [129], vitronectin [129], proteoglycans [131], elastin, entactin [132], osteonectin, aggrecan, myelin, fibrinogen [131], α1-antitripsin [133], serine protease inhibitor α1-PI, TNF precursor	
MMP-19 (RASI-1)	type I and IV collagens, gelatin [134], aggrecan [135], fibronectin, casein, laminin, nidogen [136], tenascin, cartilage oligomeric matrix protein [135]	MMP-19 activates pro-MMP-9
MMP-20 (enamelysin)	type V collagen, aggrecan [135], amelogenin [121]	
MMP-21 (Xenopus-MMP)	-	
MMP-23 (CA-MMP)	gelatin	
MMP-27 (human MMP-22 homolog)	gelatin	
MMP-28 (epylisin)	casein	

IGFB: insulin-like growth factor-binding proteins, SDF-1: stromal cell-derived factor 1, TNF: tumor necrosis factor, VEGF: vascular endothelial growth factor, mHB-EGF: heparin-binding EGF-like growth factor, CCL7/MCP: 3-chemokine (C-C motif) ligand 7/monocyte chemotactic protein 3, CX3CL1: C-X3-C motif chemokine ligand 1 and TGF beta: transforming growth factor beta.

## 3. UV Radiation as a Trigger for MMP Production

It is well-known that UV radiation causes genetic alterations in keratinocytes that are responsible for skin carcinogenesis [1]. However, there are multiple mechanisms by which UV light generates skin carcinogenesis [137,138]. There is a link between MMPs, UV radiation, skin aging and carcinogenesis [139]. Sun exposure leads to the alteration of numerous signaling pathways, such as the mitogen-activated protein kinase (MAPK), the nuclear factor-kappa beta (NF-kB), the JAK/STAT (signal transduction and activation of transcription) and the nuclear factor erythroid 2-related factor 2 (Nrf2), as critical networks for modulating inflammation and cancer [140]. Epidermal keratinocytes exposed to UVB secrete a plethora of mediators that, in turn, activate the release of MMPs by dermal fibroblasts [141,142,143]. An important role in photooxidative damage is played by proinflammatory cytokines such as interleukin (IL)-1, IL-6, IL-8 and tumor necrosis (TNF)-alpha released by irradiated keratinocytes, which will activate the MAPK pathway in dermal fibroblasts [144,145]. TNF and MMPs are important aggressiveness drivers in skin cancers. In melanoma cell lines, an aggressiveness score based on TNF and MMP-2 has been described [146]. Moreover, we have shown that the regression process in melanoma is clearly associated with a reduced tissue expression of MMPs and that TIMPs modulate their activity [147,148]. Moreover, TNF is a proinflammatory cytokine secreted by macrophages, T-lymphocytes and mastocytes, inducing an overexpression of MMP-2, MMP-3, MMP-7 and MMP-9 in the tumor microenvironment, contributing to the invasive capacity of malignant cells [149].

The protein-directed Ser/Thr kinases, extracellular signal-regulated kinase (ERK), cJun N-terminal kinase (JNK) and p38 kinase are activated by proinflammatory cytokines and further promote the expression of activator protein 1 (AP 1), which is a positive regulator of MMP production [144]. The AP-1 transcription factor results from the dimerization of Jun (c-Jun, JunB and JunD) and Fos (c-Fos, FosB, Fra-1 and Fra-2) proteins and is activated through phosphorylation under the action of various stimuli, such as growth factors, cytokines, UV radiation, etc. [150,151]. UV-induced apoptosis in dermal fibroblasts requires JNK for the release of cytochrome C from the mitochondria [152]. The P38 kinase promotes COX-2 expression and, additionally, regulates the stabilization of genes that encode for IL-8 and IL-6, inducing a proinflammatory milieu [153].

Increased AP-1 and NF-κB expression is associated with large amounts of proinflammatory cytokines, such as IL-8, that would induce tumor cell proliferation and survival in squamous cell carcinoma of the head and neck [154,155]. AP-1 promotes the transcription of certain MMPs involved in ECM degradation (MMP-1, MMP-2, MMP-3 and MMP-9) and inhibits the production of type I collagen. MMP expression and activity can be regulated at many levels (gene transcription, proenzyme activation, post-translational modifications, extracellular inhibition and degradation) [156]. Primarily, the expression of MMPs is regulated at the transcriptional level, resulting in low levels of these enzymes in physiological conditions. MMPs share cis-regulatory elements in their promoter sequences, which allow the stimulation/inhibition of their expression. There are many molecules that could modulate the MMP expression, such as hormones, cytokines and growth factors. Post-transcriptional regulatory processes have been also described to be involved in the modulation of MMP gene expression (methylation, phosphorylation, acetylation, etc.) [156,157].

UVA promotes the activation of the epidermal growth factor receptor (EGFR)-signaling pathway and the tyrosine phosphorylation of β-catenin. Subsequently, β-catenin is translocated into the nucleus and binds to T-cell transcription factor 4 (TCF-4), resulting in the stimulation of MMP gene transcription [158]. The Wnt/β-catenin pathway regulates numerous processes, including cell proliferation, migration and invasion [159]. Moreover, B-catenin mediates several genetic transcripts, such as cyclin D, matrilysin metalloproteinase, survivin, etc., involved in tumor development [160]. UVA-induced EGFR expression promotes p70 (S6K)/p90 (RSK) activation by PI-3 and ERK kinases. The activation of EGFR, in conjunction with PI-3 kinase or MAPK signaling, leads to the expression of downstream effector molecules that affect the function of certain components of the mRNA translation apparatus. The activation of the two molecules, p70S6K and p90RSK, induces AP-1 expression and promotes tumor development [161]. EGFR overexpression has been identified in various tumors, being involved in the modulation of various signaling pathways responsible for cell proliferation, invasion and metastasis. In BCC, the crosstalk between the EGFR and Hedgehog pathways induces the activation of RAS/MEK/ERK and JUN/AP-1 signaling [162].

## 4. Tumor Microenvironment—An Important Source of MMPs in BCC

MMPs are important components of the tumor microenvironment, having essential roles in cancer progression by modulating cell growth, differentiation and migration. MMPs mediate the release and activity of numerous molecules, such as cytokines, growth factors and adhesion molecules, which regulate the function of the cells encountered in the tumor microenvironment [163]. MMPs are involved in ECM degradation when the primary tumor is initiated, but correspondingly, MMPs are involved in the metastatic process when a new tumor niche in the secondary organ/tissue is established [123]. Cancer-associated fibroblasts (CAFs) actively participate in the remodeling of the ECM, and besides secreting collagen and fibronectin, CAFs actively produce MMPs (e.g., MMP-1, MMP-3, MMP-7, MMP-9 and MMP-13); these MMPs concomitantly release growth factors (e.g., VEGF) from the ECM, facilitating neoplastic cell migration together with the pro-tumoral action of CAFs [164]. At the molecular level, MMPs process adhesion and cytoskeletal proteins, favoring cancer progression [165]. The intracellular roles of MMPs should not be neglected. MMP-2 is representative for intracellular MMPs, and its roles are ancient and conserved [166]. It is important to point out that both extra- and intracellular MMPs are involved in carcinogenesis. MMP-1 was shown to promote tumor growth and chemoresistance. This can be explained by the fact that MMP-1 was identified in the mitochondrial membrane and in the nucleus and may inhibit caspase activation, conferring resistance to apoptosis [167]. In osteosarcoma cells, nucleolar MMP-2 induces ribosomal RNA transcription and proliferation of malignant cells through the cleavage of the N-terminal tail of histone H3 [16]. Nuclear MMP-3 is correlated with cancer progression in glioma. MMP-3 promotes the transcription of the connective tissue growth factor (CTGF) gene, involved in cell migration and tumor growth, by interacting with heterochromatin protein gamma [16,168]. MMP-9 accelerates the extravasation of VEGFR-1^+^ cells in the tumor niche. VEGFR-1^+^ cells will activate integrin and chymosin to promote adhesion, survival and growth in tumor cells. In the lungs of patients diagnosed with esophageal cancer, melanoma and ovarian cancer, a high expression of MMP-9 was found, which suggests that primary tumors can stimulate the production of MMP-9 in premetastatic areas [169].

**Figure 1:**
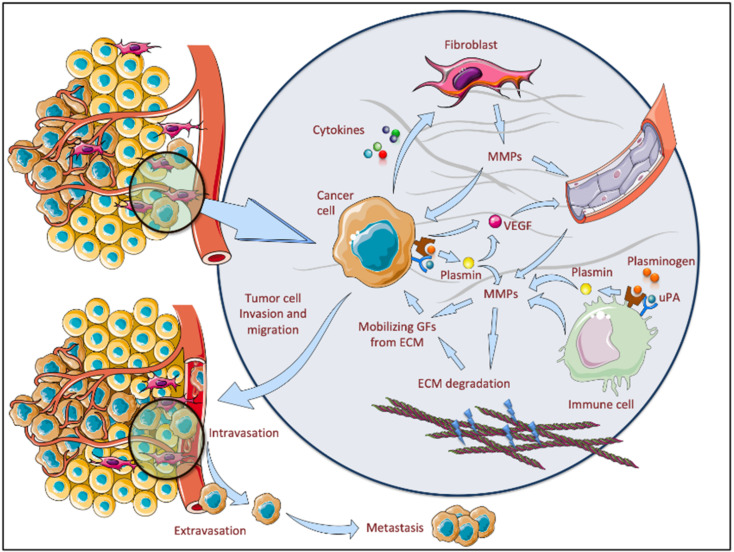
The interplay between MMPs and the tumor microenvironment. In the tumor microenvironment, MMPs are released by fibroblasts and immune cells, as well as by tumor cells. Plasminogen binds to its receptors and is converted to plasmin by urokinase plasminogen activator (uPA). In turn, plasmin may activate various MMPs and certain growth factors. All these molecules create a suitable microenvironment for ECM degradation, tumor cell invasion and migration.

Nevertheless, fibroblasts and inflammatory cells are important sources of MMPs rather than malignant cells, thus demonstrating the clear role of inflammatory infiltrates in tumor progression [170]. Plasminogen binds to its receptors and is converted to plasmin by plasminogen activators, such as the urokinase plasminogen activator (uPA). Plasmin may activate various proMMPs, including MMP-1, MMP-3 and MMP-9, and certain growth factors, such as VEGF and transforming growth factor beta (TGF-β). Therefore, plasmin has a proteolytic activity, being involved in the release of ECM components, but may also activate intracellular signaling pathways [171] (Figure 1—parts of the figure were drawn by using and modifying pictures from Servier Medical Art under https://creativecommons.org/licenses/by/3.0/, accessed on 16 June 2021). The tumor microenvironment represents the obvious results of several signaling pathway activations that further induced a plethora of cytokine and growth factor releases, promoting tumor progression, invasion and metastasis [172]. These mediators are released by stromal cells (fibroblasts, macrophages, endothelial cells, lymphocytes, etc.), as well as by neoplastic cells [173]. In the tumor microenvironment, two main types of macrophages have been identified: anti-inflammatory M1 and proangiogenic M2 polarized macrophages, with a predominance of M2 macrophages in aggressive tumors. Kaiser et al. pointed out that, in BCC, the ratio between M1 and M2 macrophages is not involved in tumor aggression, but other factors such as the expression of cyclooxygenase (COX)-2 would influence the tumor aggressiveness [174]. However, Tiju et al. observed that, in aggressive forms of BCC compared to those with milder evolution, there is a higher number of macrophages, which can induce the secretion of MMP-9 in BCC cells and enhance tumor invasion through the activation of a p38 MAPK/NF-kB/COX-2 cascade [175]. On the contrary, Padoveze et al. did not reveal a relationship between tumor-associated macrophages and recurrent BCC [176]. At least, in other cancers, in lung adenocarcinoma (LUAD), a comprehensive proteogenomic characterization of the tumors in comparison to matched normal adjacent tissues showed that, besides other molecules, MMP-8, MMP-9 and MMP-12 are correlated with rich macrophage infiltration [177]. In pancreatic cancer, a recent work has shown an association between the MMP-28 gene and immune cells infiltrating a tumor [178].

Data on tumor-infiltrating lymphocytes in BCC are scarce; however, histopathological examinations have revealed a dense infiltrate that seems to correlate with a better course of the disease [179]. CD4 + T-helper cells and FoxP3 + T-regulatory (Tregs) cells are predominant in the intra- and peritumoral inflammatory infiltrates in BCC samples; the number of CD8 + T cells, NK and immature dendritic cells is variable, depending on several factors [180]. A high amount of CD8 + T cells represents a host antitumor response, and a reduction in the number of CD8 + T cells is associated with an enhanced risk of tumor recurrence [181,182]. Additionally, an increased number of Tregs is associated with an unfavorable outcome [172]. MMPs have the ability to cleave IL-2Rα on the T-cell surface and impede their proliferation. Additionally, MMP-11 can indirectly increase the survival of malignant cells in the presence of cytotoxic molecules released by NK cells [17,183].

In the BCC stroma, as well as in the peritumoral area, numerous CAFs that exhibit morphological alterations compared to normal fibroblasts and a loss of contact inhibition are present [172]. As previously stated, CAFs can secrete various MMPs, including MMP-1, MMP-2, MMP-3, MMP-9, MMP-11, MMP-13, MMP-14 and MMP-19, which promote ECM remodeling [123]. CAFs may also be involved in tumor escapes from the immune response by releasing MMP-2, MMP-9 and MMP-14 [163].

Tumor cells mitigate the immune response by releasing MMP-2, MMP-9, MMP-13 and MMP-14, resulting in the inhibition of T-cell proliferation and antigen presentation [163]. Additionally, in BCC, there is an immunosuppressive micro medium characterized by immature dendritic cells and Th2-type cytokines [184]. In contrast, in regressing BCC, Th1-type cytokines are predominant [180].

## 5. The Role of MMPs in BCC Pathogenesis

Notwithstanding with the fact that numerous studies assessing MMP expression are available, many of them provide limited data regarding the biologically relevant activity of MMPs. Assays specifically designed to detect the activation, exposed active sites and enzymatic activity of MMPs should be employed. Crawford et al. employed fluorescent MMP substrates (both in vitro and in vivo) to characterize the patterns of MMP activity in zebrafish embryos. By using conventional gelatin zymography, MMPs were identified in the embryos as early as 3 somites, whereas in vivo techniques revealed type IV collagen degradation at the somite boundaries in as early as 4 somites [185]. The association between immunostaining and in vivo activity-based protein profiling (“a method that uses a chemical probe that targets active MMPs and becomes covalently bound to the protease”) can characterize MMP localization and MMP activity [186]. Most studies evaluating MMP activation are based on tissue culture and biochemical assays. However, the native tissue context influences the activity of MMPs [187].

Significant progress has been made in deciphering the pathogenesis of BCC, but there are still numerous gaps in understanding the processes involved in the occurrence and evolution of BCC. In recent years, numerous studies have deepened our understanding of the role of MMPs in BCC.

### 5.1. The Expression of MMPs in BCC

A study conducted by Yucel et al. suggested that MMP-1 plays the most important role in the degradation of collagen in BCC [188]. The degradation of intact type I collagen results in high molecular weight collagen fragments; these fragments are not further degraded, and as MMP-2 and MMP-9 continue to degrade the intact collagen, high molecular fragments accumulate in a high rate. The accumulation of high molecular weight collagen fragments impairs the function of stromal fibroblasts. These events may underlie the increase in MMP production associated with BCC development [188]. When the intact collagen is fragmented, high molecular weight breakdown products are biologically active. Fibroblasts in contact with large collagen fragments change from a pro-synthesis phenotype that implies increased procollagen synthesis in association with a low MMP expression to a degradative phenotype characterized by an increased MMP expression and a reduced collagen synthesis [188,189]. The high expression of MMP-1 in the tumor stroma induces structural changes at the tumor periphery, with a loss of the palisading arrangement, which suggests a poor differentiation and a histological aspect that is correlated with an unfavorable prognosis [190].

Chen et al., using RT-PCR, evaluated the expression of MMP-2 in independent cultures and observed that MMP-2 is overexpressed in fibroblasts and melanoma cells and, to a much lesser extent, in keratinocytes and BCC cells. In contrast, when the noncontact cocultivation of fibroblasts with keratinocytes or BCC cells was performed, a decrease in MMP-2 expression by fibroblasts was observed. When the cocultivation with melanoma cells was performed, MMP-2 expression by fibroblasts was increased. Performing the same steps for MMP-1, noncontact cocultivation with keratinocytes, BCC cells and melanoma cells led to an increased expression of MMP-1 by fibroblasts. These results suggest the role of epidermal–mesenchymal interactions and the host immune response in the progression of BCC [191].

Monhian et al. showed a higher expression of MMP-1 and MMP-9 in the peritumoral tissue compared to the areas of the skin located distal to the tumor and a significant correlation between the presence of active gelatinolytic enzymes and broad fragmentation of the collagen substrate. Several factors could be involved in the increased MMP expression in tumor-associated tissue. EGFR agonists such as heparin-binding epidermal growth factor and amphiregulin could promote cell proliferation and stimulate MMP expression. A role was also attributed to proinflammatory cytokines such as IL-1, a significant amount of IL-1 identified in the skin, which acts as an inducer of gene transcription for MMPs [192]. Manola et al. evaluated the role of MMP-2 and MMP-9 in the peritumoral cleft present in the BCC samples, but they did not find a statistically significant correlation between the MMP expression and the presence of the peritumoral cleft [193]. Varani et al. showed, by analyzing 54 histological samples of BCC, different patterns of MMP expression. MMP-1 and MMP-2 were expressed both in tumor cells and normal epithelial cells, but their activity was higher in BCC compared to normal skin. MMP-8 was expressed only in the stroma. In contrast, MMP-9 and MMP-13 were expressed mainly in the healthy skin adjacent to the tumor [189]. Zlatarova et al. conducted a study on eyelid BCCs that revealed an increased expression of MMP-1, MMP-9 and MMP-13 in malignant epithelial cells but, also, in the surrounding stroma (inflammatory cells, fibroblasts and endothelial cells). Moreover, a significantly increased expression of MMPs was detected at the periphery of the tumor, an area with local invasion potential [194]. Petterson et al. showed that the depletion of cell surface CD44 was correlated with a high MMP-7 expression in BCC and SCC samples [195].

The study reported by Ciążyńska et al. was the first one to highlight the MMP-8 expression in BCC, using RT-PCR and a Western blot analysis. They identified the overexpression of MMP-8 mRNA in BCC; however, the Western blot analysis revealed only a slight increase in the MMP-8 protein expression. The study also showed important differences in the levels of mRNA and protein overexpression of MMP-1, MMP-3 and MMP-9 in the tumor tissue compared to normal skin [196].

Hattori et al. revealed that MMP-13 was expressed by the endothelial cells in 17 of the 20 BCC analyzed samples, at both mRNA and protein level, whereas MMP-1 was identified in only two cases. MMP-13 was also detected in the microvessels of normal skin from the edge of the surgical wound. These data suggest the role of MMP-13 in angiogenesis and indicate that the endothelial cells in the skin represent a significant source. The study pinpointed that MMP-13 expression is upregulated by IL-1alpha and, to a lesser extent, by phorbol myristate acetate, a tumor promoter, and by tumor necrosis factor alpha [197]. El-Havary et al. immunohistochemically evaluated the expression of MMP-13 and the cellular marker of Ki 67 proliferation in BCC and SCC specimens from patients with and without xeroderma pigmentosum. They did not observe differences in the MMP-13 expression between the two groups, but when analyzing the Ki67 expression, they detected higher levels in those with xeroderma pigmentosum, which may explain the more aggressive behavior of these tumors in patients with xeroderma pigmentosum [198].

Boyd et al. compared the expression of several MMPs (MMPs-1, -7, -8, -9, -10, -13 and -26) and their tissue inhibitors (TIMPs-1 and -3) in BCC samples from kidney transplant recipients and a control group of immunocompetent individuals. The immunohistochemical analysis did not reveal significant differences between the two groups regarding the expression of MMPs by BCC cells. However, MMP-1, MMP-9 and TIMP-1 were more frequently expressed by stromal macrophages in BCC samples from immunocompetent individuals, emphasizing the role of the tumor microenvironment in BCC behavior in association with the patient’s immune status [199].

### 5.2. The Link between MMPs and BCC Invasiveness and Recurrence

The release of MMPs is one of the first events in the complex process of tumor invasion, resulting in important cytoskeleton changes that will allow cell migration. This process is governed by molecular interactions, cell-to-cell adhesion molecules such as E cadherin and β-catenin and chemokine receptor ligands such as CXCR4 [6]. MMPs could play a role not only in the aggressiveness of the tumor but, also, in its recurrence.

Histopathologically, BCC can be divided into six types: nodular, superficial, infiltrative, morpheaform, micronodular and mixed. The most common types encountered in medical practice are the nodular and superficial BCCs [179,200]. Based on its invasive behavior and recurrence risk, BCC can be considered as high-risk BCC (the morpheaform, infiltrative or basosquamous types) and low-risk BCC (the nodular and superficial types) [201,202]. Thus, depending on the histopathological type, BCC can be considered a more aggressive or less aggressive tumor. However, in many cases, areas with aggressive growth patterns and areas with a less aggressive pattern have been present in BCC samples [203].

Poswar et al. revealed that the protein expression of MMP-2 in the tumor stroma was more extensive in high-risk BCCs compared to low-risk BCCs [201]. In line with this, the same study showed that the protein expression of MMP-2 was higher in SCC (parenchyma and stroma) compared to BCC, suggesting that MMP-2 could play a defining role in the invasive nature of tumors [201]. However, a recent study did not find differences regarding the expression of MMP-2 mRNA in infiltrative BCC compared to nodular BCC. Interestingly, a higher expression of mRNA for MMP-2 and a lower expression of mRNA for type IV collagen were observed in the tissue adjacent to the nodular type compared to the infiltrative one. In the infiltrative type, the level of type IV collagen mRNA was increased in the surrounding tissue, probably as a defense mechanism against tumor infiltration [204]. Lower amounts of type IV collagen, a major component of the basement membrane, were identified in SCC samples compared to BCC, which may explain the higher invasiveness of SCC and can be considered as a marker of aggressiveness [105,205]. A study conducted by Orimoto et al. did not show significant differences in MMP-2 mRNA expression when compared between the nodular, superficial and sclerosing BCC types; however, they suggested that MMP-2 could be regarded as a marker for the differentiation between BCC and the surrounding normal tissue [206].

Zhu et al., using an immunohistochemical analysis, showed an increased expression of MMP-9 in both primary and metastatic SCC compared to BCC and normal skin tissue, highlighting the role of MMP-9 in tumor invasion and metastasis: MMP-9 degrades collagen and elastin, allowing tumor cell migration [207]. Gozdzialska et al. detected a higher expression of mRNA for MMP-9 in infiltrative BCC compared to nodular BCC [204]. Kadeh et al. analyzed the MMP-10 expression in BCC compared to SCC samples and observed a higher MMP-10 expression in both the tumor epithelium and stroma in SCC. In addition, in the case of SCC, there was a positive correlation between the MMP-10 expression and tumor grade. The authors concluded that MMP-10 may have a role in the different invasive patterns observed in BCC and SCC, contributing to the tumor aggressive behavior [208]. Cribier et al. indicated that MMP-11 expression is elevated in high-risk BCC [209]. Consistent with this, Greco et al. detected that HMGA1 and MMP-11 mRNA expressions were higher, as well as the HMGA1 and MMP-11 protein expression levels in the BCC and SCC samples compared to healthy tissue, and the levels of the two markers were higher in SCC compared to BCC, suggesting their involvement in tumor aggressiveness [210]. Greco et al. proposed that MMP-11 as a differentiation marker between self-limiting skin tumors and more aggressive ones in nonmelanoma skin cancer [210].

Kerkela et al. detected MMP-14 mRNA in stromal fibroblasts in fibrosing and keratotic BCC samples but not in adenoid BCC samples, being the first study to evaluate the expression of this MMP in BCC. In addition, MMP-10 was expressed only in epithelial laminin 5-positive cancer cells [39]. Laminin 5, after being cleaved by MMP-2 and MMP-14, promotes cell migration and invasion [105]. In the study by Kerkela et al., the release of laminin 5 by tumor cells was observed only in sclerodermiform BCC [105]. Oh et al. suggested that MMP-14 should be considered as a marker for high-risk BCC. The mechanism by which MMP-14 could be involved in the pathogenesis of high-risk BCC is the degradation of the E-cadherin/β-catenin complex and the activation of other MMPs, such as MMP-2 [211]. Oh et al. attributed an important role to β-catenin in increasing MMP-14 expression [211]. MMP-14 degrades type I collagen, the most abundant ECM component, and modulates cell–ECM interactions, promoting cell migration and tumor invasion [27]. ECM proteolysis mechanisms are involved in regulating epithelial cells, as well as carcinoma cell trafficking in vivo. MMP-14 was identified as an important effector of the matrix-remodeling processes in breast cancer. Feinberg et al. showed that MMP-14 regulates physiologic processes such as normal mammary gland branching morphogenesis, but also, MMP-14 induces local invasion and metastasis, and they concluded that there is a differential regulation of the normal and malignant mammary epithelial cell invasion programs [212].

MMPs participate in the invasion process by degrading E cadherin, a molecule with a pivotal role in cell–cell adhesion in epithelial tissues [213]. The role of the E-cadherin/β-catenin protein complex in mesenchymal–epithelial transition is well-known and is strongly implicated in cancer progression [214].

Rogosic et al. performed a study on 64 BCC samples and evaluated the expression of MMP-1, MMP-2, MMP-9, MMP-13 and E-cadherin using immunohistochemical staining. They revealed that the expression of MMP-1 in tumor cells was five times higher in morpheaform and recurrent BCC than in superficial, cystic or micronodular BCC. In addition, the expression of MMP-9 and MMP-13 in stromal cells was associated with morpheaform and recurrent BCC. Moreover, in morpheaform and recurrent BCC, E-cadherin was absent [215].

Chu et al. analyzed 19 recurrent BCCs and observed an increased expression of CXCR4. The study showed that stromal cell-derived factor 1a (SDF-1a), the CXCR4 ligand, is involved in the invasive behavior of BCC. This mechanism is based on the upregulation of mRNA expression and activity of MMP-13 by SDF-1a through the phosphorylation of ERK1/2 and induction of the expression of the AP-1 component c-Jun [216]. CXCR4-transfected BCC cells were administered to nude mice that developed aggressive BCC with an increased expression of CXCR4 and MMP-13 [216]. Ciurea et al. investigated the expression of CXCR4, MMP-13 and β-catenin in samples of facial BCC as following: metatypical (six cases), infiltrative–morpheaform (eight cases), micronodular (six cases) and superficial (five cases). CXCR4 expression was the highest in metatypical BCC compared to the other subtypes, especially in the areas with squamous transformation. The same observations were made for MMP-13 and β-catenin. In all cases, MMP-13 expression was higher in the stroma (fibroblasts and inflammatory cells) compared to tumor cells [160]. As previously acknowledged, MMP-13 can be regarded as a marker of malignant transformation in keratinocytes and has a role in tumor invasion by degrading ECM [199].

El-Khalawany et al. performed a study on 22 samples of recurrent BCCs and analyzed the expression of COX-2, ezrin—a cytoplasmic peripheral membrane protein—and MMP-9 compared to nonrecurrent BCCs. COX-2 expression was identified in 90.9% of recurrent BCCs and 59.1% of nonrecurrent BCCs, the found difference being statistically significant. In contrast, regarding the expression of ezrin and MMP-9, there were no statistically significant differences between the two groups [217]. Karahan et al. evaluated 30 BCC samples and did not observe any significant differences between the histological types of primary BCCs with respect to MMP-2 and MMP-9 expression. Moreover, the differences were not significant between the primary BCC and recurrent BCC. However, a positive correlation was identified between the COX-2 and MMP-9 expression. The COX-2 expression was higher in recurrent BCC compared to primary BCC [218].

## 6. TIMPs—Tissue Inhibitors of Metalloproteinases—Potential New Agents in the Management of BCC

TIMPs, endogenous inhibitors of MMPs that bind to their catalytic site, represent a family of polypeptides that includes four members: TIMP-1 TIMP-2, TIMP-3 and TIMP-4 [219].

The balance between TIMPs and MMPs plays an important role in cell homeostasis and in the ECM remodeling process. The destruction of this balance leads to the appearance of numerous pathological processes involved in various diseases, including malignant tumors [220]. Structurally, TIMPs are composed of a large N-terminal domain that inhibits MMP function and a smaller C-terminal domain. The N-terminal domain of TIMPs is able to bind the majority of MMPs, while the C-terminal site of TIMP-1 and TIMP-2 binds to the hemopexin domain of pro-MMP-2 and pro-MMP-9 [21,219]. The tissue expression of TIMPs is both constitutive and inducible and is modulated by various molecules and growth factors [221]. In addition to their role in maintaining ECM homeostasis, TIMPs have many functions independent of metalloproteinases, being multifunctional proteins. They are involved in cell migration, exhibit pro and antiapoptotic activity, are antiangiogenic factors, etc. Recent studies have shown that TIMPs, in addition to their inhibitory effect on MMPs, exert an inhibitory effect on integrin-metalloproteinases, ADAMs and ADAMTSs [222].

Of note, only a few studies in the literature have evaluated the expression of TIMPs in BCC. The expression of TIMP-1 and TIMP-2 was detected in stromal cells, and TIMP-3 expression in tumor epithelial cells, in the infiltrative area [105]. Regarding TIMP-1, an increased expression was observed primarily in morpheaform BCC, which was associated with an unfavorable prognosis. Furthermore, an association between the increased expression of TIMP-1 in tumor cells and/or stroma and the recurrence rate of eyelid BCC was identified [194]. TIMPs prevent tumor development by forming a fibrotic capsule and by inhibiting angiogenesis; however, it should be emphasized that TIMPs can also act as activators of MMPs; therefore, in some cancers, there may be an increase in the expression of MMPs in association with an increase in the expression of TIMPs [194,223]. Fu et al. revealed a higher expression of MMP-2 and MMP-9 and a lower expression of TIMP-1 and TIMP-2 in SCC compared to BCC [224]. Regarding TIMP-3 expression, no differences were observed when the samples of BCC, SCC and actinic keratoses were analyzed [201].

Recent research has suggested that TIMPs could be used as therapeutic agents in carcinogenesis and influence the outcome and prognosis of neoplasms [223].. Mutagenesis studies have revealed mutations that could improve the selectivity of TIMPs for MMPs. There are some studies with encouraging results; for instance, the affinity of TIMP-1 toward MMP-14 was increased by replacing a single amino acid in the binding interface [225]. In addition, in another study of selective MMP-14 inhibition, the authors created a mutant TIMP that blocked the collagenase activity of MMP-14 in cell culture models of breast cancer and fibrosarcoma [226].

## 7. Conclusions

Most of the studies in this field show that MMPs are overexpressed in BCC, where they promote the release of numerous molecules, such as cytokines, growth factors and adhesion molecules, which provide a favorable environment for tumor development and progression. The majority of the studies investigating MMPs in BCC are correlative analyses of expression data that is often based on mRNA levels or protein levels, and very few studies have examined the biologically relevant activity of these proteases in the context of their possible roles in BCC. Knowledge of the MMP profile in BCC samples could be the basis for predicting the characteristics of tumor behaviors, particularly with respect to aggressiveness and amenability to specific clinical interventions. However, data on the substrates of these enzymes in vivo and their roles in processes other than ECM remodeling are scarce; therefore, correlations between MMP expression and tumor aggressiveness are difficult to interpret. This review gathered up and organized the newest studies on the matter, an endeavor that has not been undertaken in recent years, although data on the MMP involvement in BCC has been building up. Based on the aforementioned studies, we believe that MMP-targeted therapies could find their place in BCC management; however, further studies are needed.
